# Transient cytotoxicity of ^131^I beta radiation in hyperthyroid patients treated with radioactive iodine

**Published:** 2011-04

**Authors:** P. Shanmuga Sundaram, S. Padma, S. Sudha, K. Sasikala

**Affiliations:** Department of Nuclear Medicine, Amrita Institute of Medical Sciences, Cochin, India; * *Department of Biotechnology, Karpagam Academy of Higher Education, Karpagam University, Coimbatore, India*; ** *Department of Zoology, Bharathiar University, Coimbatore, India*

**Keywords:** chromosomal aberrations, hyperthyroidism, low dose ^131^I therapy, micronucleus assay

## Abstract

**Background & objectives:**

Radioiodine (^131^I) or radioactive iodine in low doses is used worldwide as the first line of management in the treatment of hyperthyroidism. Information is available on the extent and severity of cell damage after a high dose radioiodine (^131^I) therapy for thyroid cancer, but information is scanty on its cellular effects, its extent and severity of cell damage after a low dose ^131^I therapy. The present investigation was aimed to study the cytotoxic effects of a low dose ^131^I therapy in varying doses as is normally being used in routine clinical practice in the treatment of various forms of hyperthyroidism.

**Methods:**

Peripheral blood lymphocytes were analyzed in 32 hyperthyroid patients. All of them received ^131^I in the form of sodium iodide solution orally. Blood lymphocytes were studied for the presence of chromosomal aberrations (CA) and micro nucleus (MN) using micronucleus assay. Blood samples of these patients were drawn prior to the treatment, on 7 ^th^and 30 ^th^days after the treatment.

**Results:**

The results indicated a positive relationship between ^131^I dose, CA and MN frequency. A statistically significant increase in CA and MN frequency in day 7 post- therapy and a decrease in mean levels of CA and MN on day 30 post-therapy were observed when compared to pre-therapy.

**Interpretation & conclusions:**

This study showed that the cytogenetic damage induced by ^131^I in low doses i.e., less than 555MBq was minimal and reversible. Patients can be motivated to undertake this safe and easy procedure as a first line of therapy in the treatment of hyperthyroidism.

The use of radioiodine ^131^I has continued to remain as mainstay of therapy for hyperthyroidism including Graves′ disease, toxic multinodular goiter and autonomous toxic nodule. Despite the advantages of beta radionuclide therapy in destroying the over-functioning thyroid tissue with ^131^I, side-effects can also occur in these patients because normal tissues are simultaneously exposed to beta radiation. In all hyperthyroid patients treated with ^131^I, the optimal outcome is obviously euthyroidism without post ablative hypothyroidism but one cannot reliably accomplish this goal considering the number of variables affecting the outcome, including characteristics of the patient (*i.e*., age, gender, and gland size); severity and duration of the underlying autoimmune thyroid stimulus; radiation delivered to the gland (*i.e*., ^131^I fractional uptake, homogeneity of distribution, and effective half-life); and preceding antithyroid drug therapy. There is an almost linear relation between the amount of administered radioiodine and the development of subsequent hypothyroid disease. Administration of smaller amounts of Iodine-131, however, increases the chances of treatment failure and the need for repeated radioiodine administration. Because most treated patients with Graves’ disease (regardless of treatment used) ultimately become hypothyroid, it is better to tell patients to expect this as an acceptable late result of treatment^1^. Complications of radioiodine therapy for hyperthyroidism are transient, minimal and usually related to gastrointestinal disturbances like altered taste, nausea and vomiting. The growing awareness of subtle short- and long-term consequences of this therapy outweighs the potential therapeutic benefit of this one time therapy.

^131^I is a reactor-produced radioisotope with a physical half-life of 8.02 days. It has both beta and gamma radiation components which can be harmoniously used for therapeutic and diagnostic benefit. On oral administration it is rapidly and completely absorbed from stomach. The beta emission from radioiodine disrupts chemical bonds throughout the cell, inflicting devastating damage on the DNA molecule and triggering cellular dysfunction and ultimately death of thyroid follicular cells^2^,^3^. It produces chromosomal aberrations like breaks, deletions through its beta emitting properties. Asli *et al*^4^ studied the cytogenetic effects in patients exposed to Technetium during cardiac imaging and radioiodine during thyroid cancer therapy and found the cellular changes to be statistically insignificant. ^131^I treatment takes advantage of the fact that thyroid cells have the ability to absorb iodine after oral administration due to the presence of an active sodium iodide symporter. There is normal physiological uptake of ^131^I in salivary, lacrimal gland, nasopharynx and lactating breast also.

The cellular effects of low levels of ionizing radiation can be studied using cytogenetic assays^5^,^6^. Peripheral blood lymphocytes are best suited for studying the effects of radiation as these are the most sensitive cells to ionizing radiation^7^,^8^. The present study was planned with the objective of estimating the cytogenetic effects of a low dose ^131^I therapy in the treatment of various forms of hyperthyroidism in varying doses.

## Material & Methods

*Samples:* The study included a total of 32 hyperthyroid patients (M: F = 14: 18), age range between 23-61 yr, mean 49 ± 7 yr who were under evaluation in Endocrinology department of Amrita Institute of Medical Sciences, Cochin, Kerala. Clinically hyperthyroid patients were sent to Nuclear medicine department of the same Institute where a Technetium thyroid scan was performed. Patients were selected consecutively from June 2007 to December 2008. Based on the thyroid scan findings of Graves′ disease, toxic multinodular goiter and autonomous toxic nodule empirical ^131^I dosage was decided as per the standard treatment protocol^9^. All patients were clinically and biochemically evaluated prior to Technetium thyroid scan by the endocrinologist. Basic blood tests along with serum free T4, T3 and TSH estimations were performed on their initial presentation to the endocrinologist. Control groups *i.e*., volunteers (who came for routine health checkup) (20 in number) were enrolled for this study. All of them were clinically normal with normal thyroid function tests and Technetium thyroid scan. They were not administered ^131^I and their blood samples were kept as control. All subjects were informed of the objective of the study and gave their consent. The institutional ethical committee approved the research procedures used in this study.

*Inclusion criteria:* Clinical, biochemical and scintigraphic confirmation of hyperthyroidism as Graves’ disease, toxic multinodular goiter and autonomous toxic nodule. Scintigraphic confirmation was done by Technetium thyroid scintigraphy in patients who were off antithyroid drugs for atleast five preceding days.

*Exclusion criteria:* (*i*) Scintigraphic diagnosis of subacute viral thyroiditis, (*ii*) pregnant women and those breastfeeding who could not wean off baby for eight weeks post-treatment with radioiodine, (*iii*) patients with free T4 levels > 5 ng/ml at the time of treatment were excluded as ^131^I administration may lead to a medical crises called thyroid storm with excess release of preformed hormones into circulation and (*iv*) patients with Graves’ ophthalmopathy.

The treatment consisted of ^131^I administration orally as an adjuvant radiation dose to destroy thyroid follicular cells. All patients recruited for this study were explained the details of the treatment, radiation safety measures to be followed for 10 days after the treatment. They were also instructed to stop iodised salt, seafoods and iodide containing medications atleast 3 weeks prior to this treatment. Antithyroid medications like carbimazole was stopped a week prior to the date of therapy.

Based on the trapping function of thyroid gland established by Technetium scan, patients were divided for empirical ^131^I dosage into 185 – 370 MBq, 370 – 555 MBq and above 555 MBq of ^131^I. Twenty five patients with Graves′ disease (GD), received a dose of 185 –370 MBq, five patients of toxic multinodular goiter (TMG) received between 370 – 555 MBq and remaining two patients of autonomous thyroid nodule (ATN) were administered more than 555 MBq of ^131^I therapy. Five ml of heparinised blood were collected from all these patients just before administering ^131^I low dose. Subsequent blood samples were collected from each patient one week and 30 days after the therapy to look for DNA changes.

*Chromosomal analysis:* RPMI1640 (Gibco, USA) foetal calf serum (Gibco) phytohaemagglutinin – M (Gibco) dimethyl sulphoxide 5 μl /ml (E.Merk, India) colchicines 0.20 μg/ml (Micro lab) and cytochalasin – B (Sigma, USA) were used for this analysis.

Peripheral blood cultures were prepared^10^,^11^. Blood sample (0.5 ml) was inoculated under aseptic conditions into a culture vial containing 5.0 ml of culture medium, 1.0 ml of AB serum (Antibodies Inc. Washington, DC) and 0.2 ml of phytohaemagglutinin. The cultures were incubated at 37°C for 72 h. The dividing cells were arrested at the metaphase stage by adding 0.05 ml of colchicines solution (0.01%) 30 min before harvesting the culture. The contents of the vials were centrifuged at 200 g for 10 min at the end of colchicine treatment. The supernatant was discarded and 6 ml of hypotonic solution was added to the test tube after disturbing the cell button. The contents of the test tubes were incubated for 7 min and freshly prepared fixative (Methanol: glacial acetic acid 3:1v/v) was added and centrifuged at 200 g for 10 min. Later the supernatant was discarded and two or three changes of fixative were given to obtain colourless cell pellet. The slides bearing chromosome spreads were treated with 0.25 per cent trypsin for 3 to 10 sec and were stained in 4 per cent buffered Giemsa solution for 3 min. At least 100 metaphases were examined for the occurrence of different types of abnormality *i.e*., gaps, fragments, breaks, *etc*. Criteria to classify the different types of aberrations were in accordance with the recommendation of Environmental Health Criteria 46 for environmental monitory of human population^12^.

*Micronucleus analysis:* The lymphocytes were cultured according to the method of Fenech and Morley^13^. Pokeweed mitogen (Gibco BRL, USA) was used to stimulate the lymphocytes to proliferate in culture. A solution of cytochalasin B (Aldrich Chemical Co., India) was added 44 h after the commencement of the culture. The cultures were terminated 72 h after initiation. Scoring of MN was limited to bi-nucleated lymphocytes with preserved cytoplasm according to the criteria proposed by Countryman and Heddle^14^. The following are the criteria used for scoring the cell inclusions: (*i*) only cells with a distinct cytoplasm and distinct binucleation were analyzed for the presence of MN, (*ii*) only MN entirely inside the cytoplasm, separated from the main nucleus and with a diameter <1/3 of the size of the main nucleus were scored, and (*iii*) only MN staining the same as or lighter than the nucleus were scored. The suggested criteria for identifying MN were; (*i*) rounded smooth perimeter suggestive of a membrane; (*ii*) less than a third the diameter of the associated nucleus, but large enough to discern shape and colour; (*iii*) Feulgen positive, i.e., pink in bright field illumination; (*iv*) staining intensity similar to that of the nucleus; (*v*) texture similar to that of nucleus; (*vi*) same focal plane as nucleus; and (*vii*) absence of overlap with, or bridge to, the nucleus. The results are expressed as the average percentage of micro nucleated cells per binucleated cells.

**Fig. 1:**
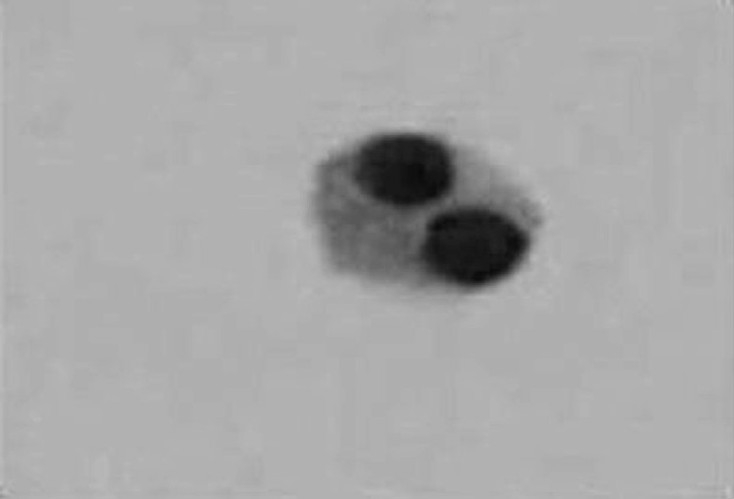
Binucleate cell with no micronuclei in a patient of Graves’ disease treated with 10 millicurie of ^131^I.

**Fig. 2:**
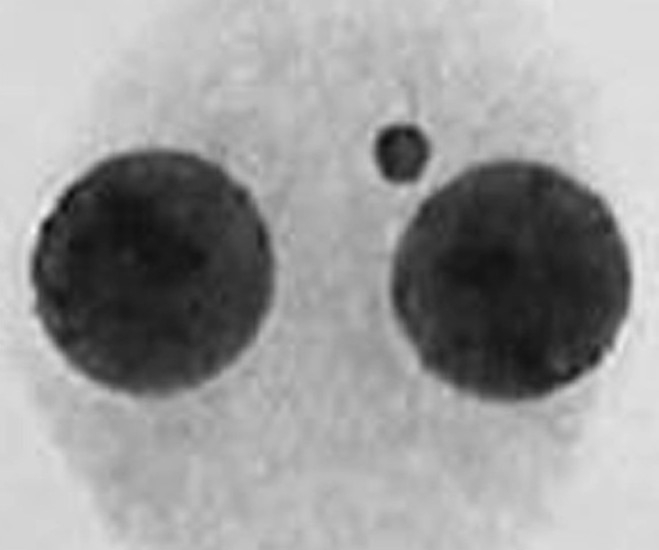
Micronucleus in a binucleate cell of a patient with toxic multinodular goitre treated with more than 10 millicurie of ^131^I.

**Fig. 3:**
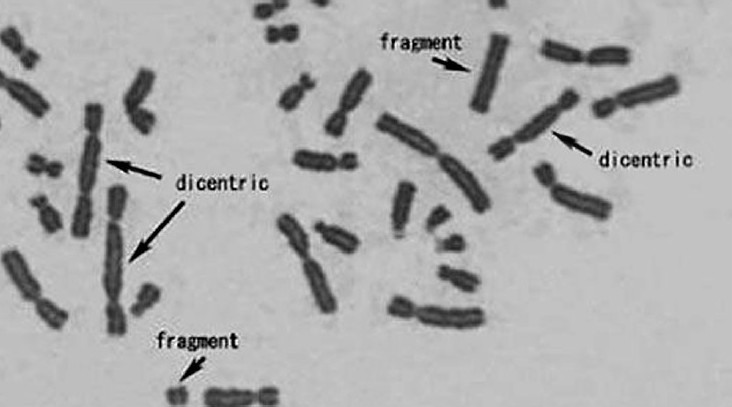
Chromosomal aberrations seen in a patient with autonomous toxic nodule treated with 15 millicurie of ^131^I.

Metaphases were scored for CAs and included chromatid gaps, isochromatid gaps, breaks, fragments, **etc**. Gaps are true discontinuities of the chromosome structure^15^ and in this study metaphases were studied with and without gaps (Figs 1–3).

*Statistical analysis:* Various statistical methods (Student’s *t*-test, Mann–Whitney test, Kolmogorov–Smirnov test and Pearson′s rank correlation analysis) were used depending on the nature of the data and the type of analysis needed. SPSS 11.01 statistical software (SPSS Inc., Chicago, IL) was used for the calculation of the results obtained.

The association of the frequency of micronuclei with the age and sex of subjects was tested with Pearson’s rank correlation analysis.

## Results

In our study, female hyperthyroid patients were able to tolerate symptoms and signs for 1 to 2 years when compared to males, who had lower tolerance levels of around 6 months to 1 year. The predominant symptoms were weight loss followed by increased appetite, tremors, increased sweating, palpitations and mental disturbances.

In cytogenetic study, there was a moderate increase in chromosomal aberrations (CA) in day 7 post ^131^I therapy blood samples when compared to the pretreatment samples. A significant decrease of the same was observed in the day 30 post ^131^I therapy samples (Table I). Lymphocytes of control groups showed fewer MN and CA when compared to sample I (185-370 MBq).

**Table I:** Frequency of chromosomal aberrations (CA) in control population and in ^131^I-treated hyperthyroid patients 7 and 30 days after therapy

Treatment dose (MBq)	Number of subjects (N=20)	Abnormal metaphases		Chromosome aberrations			
		Number	Frequency	Gaps		Fragments and breaks	
				Number	%	Number	%
Control	20	2	0.10	1	0.15	0	0
Sample I							
185-370	25	5	0.60	2	0.25	5	2.09
370- 555	5	7	0.53	3	0.98	6	1.98
>555	2	6	0.66	3	1.00	6	2.00
Sample II							
185-370	25	4	1.24	2	1.03	4	1.68
370- 555	5	6	2.03	2	0.56	4	1.67
>555	2	6	2.64	2	0.54	4	1.46
Sample III							
185-370	25	4	1.00	2	1.03	4	1.68
370- 555	5	6	1.67	2	0.56	4	1.67
>555	2	6	2.85	2	0.54	4	1.46

Sample I, Pre-therapy control groups for 3 subsets of patients; Sample II, day 7 post-therapy; Sample III, day 30 post-therapy

In MN analysis, a moderate increase in micronuclei was observed in patients treated with higher dose of ^131^I. Similarly there was a transient increase in the number of MN (Table II). in day 7 compared to day 30 post-therapy samples. Patients receiving higher dosage of ^131^I showed both MN and abnormal metaphases in their blood samples. However there was no clear correlation between the micronuclei frequency and the therapeutic ^131^I dose used. In addition, age and sex did not show any influence on micronuclei frequency in either patients or control population (r=0.04, *P*>0.05).

**Table II:** The Frequency of Micronuclei (MN) observed in ^131^I treated hyperthyroid patients- 7 & 30 days after therapy

Disease Particulars	Age in years (Mean ± SD)	Frequency of Micronuclei (Mean ± SD)	
		7 days after ^131^I therapy	30 days after ^131^I therapy
GD (n= 25)	46.32 ± 6.68	2.36 ± 1.380	1.88 ± 0.175*
TMG (n=05)	55.00 ± 8.24	4.60 ± 0.96	3.30 ± 0.97*
ATN (n=02)	53.5 ± 6.36	6.00 ± 0.00	4.25 ± 0.35*

* Values insignificant at 5% level; GD, graves’ disease; TMG, toxic multinodular goiter; ATN, autonomous thyroid nodule.

Both MN and abnormal metaphases seen in hyperthyroid patients treated with ^131^I was insignificant when compared to control group.

## Discussion

The results revealed that low dose ^131^I therapy induce chromosomal abnormalities in the form of increased CA and MN as early as 7 days after treatment. Similar observations on chromosomal abnormalities and structural aberrations in ^131^I hyperthyroid subjects have been reported earlier^16^. Ramirez *et al*^17^ reported a 1.8 fold increase in the frequency of 17p breaks after iodine-131 therapy in hyperthyroid patients. There is a minimal cytogenetic damage in the peripheral blood lymphocytes of ^131^I treated hyperthyroid patient in comparison with controls in their study. Similar results were observed by Guitizeez *et al*^18^.

Micronuclei are small nuclei like particles seen apart from the nucleus in an irradiated cell. These enclose acentric fragments or whole chromosomes that have not been included in the main nuclei at cell division and in the cytoplasm^19^. Therefore, the presence of micronuclei can indirectly denote chromosome breakage or impairment of the mitotic spindle. It has been shown that ionizing radiation induces micronuclei in human lymphocytes and, in spite of the variability of dose-response relations; there is a quantitative relationship between radiation dose and frequency of micronuclei, which can be used for biological dosimetry. The sensitivity of cytokinesis blocked micronuclei assay is well established^20^.

In our study, it was observed that the increase in micronuclei after therapy, was not statistically significant which is explained by the fact that the amount of radiation induced to cells may be minimal. Watanebe *et al*^21^ in their study reported an increase in MN after ^131^I therapy and they found that number of MN peaked at 3 days after ^131^I administration and gradually decreased.

Radiation induced ionization may act directly on the cellular component molecules indirectly or water molecules causing formation of water derived radicals. Radicals react with nearby molecules in a very short time resulting in breakage of chemical bonds or oxidation of the affected molecules. The DNA is a major target for radiation damages because DNA distraction can kill or mutate human cells. Double strand DNA breaks may play a major role in these biological effects^8^,^22^. The relatively low frequency of chromosomal aberrations and MN induced by ^131^I *in vivo* supports the contention that cytogenetic damage of this therapy in hyperthyroid patients was minimal transient and reversible.

To conclude, human peripheral blood lymphocytes are sensitive to ionizing effects of radiation, and low dose ^131^I therapy induces transient chromosomal abnormalities in the form of increased CA and MN in patients with hyperthyroidism as early as 7 days after treatment, which (*P*>0.5) reduces 30 days following therapy. These patients can be motivated to undertake this safe and easy procedure as a first line of therapy in the treatment of hyperthyroidism.

## References

[1] Maxon HR, Smith HS (1990). Radioiodine-^131^ in the diagnosis and treatment of metastatic well differentiated thyroid cancer. *Endocrinol Metab Clin North Am*.

[2] Howarth D, Epstein M, Lan L, Tan P, Booker J (2001). Determination of the optimal minimum radioiodine dose in patients with Graves’ disease: a clinical outcome study. *Eur J Nucl Med*.

[3] Sgouros G, Kolbert K, Sheikh A (2004). Patient-specific dosimetry for ^131^ I thyroid cancer therapy using 124 I PET and 3-dimensional-internal dosimetry (3D-ID) software. *J Nucl Med*.

[4] Asli IN, Mosaffa N, Mogharrabi M, Hooman A, Tabei F, Javadi H (2010). Chromosome aberrations after high dose I 131 and 99mTc MIBI administration. *Nucl Med Commun*.

[5] Blackwell N, Stevenson AC, Wiernik G (1974). Chromosomal findings in patients treated with small doses of iodine-131. *Mutat Res*.

[6] Fenech M (2000). The *in vitro* micronuleus technique. *Mutat Res*.

[7] Gutiérrez S, Carbonell E, Galofré P, Creus A, Marcos R (1999). Cytogenetic damage after 131-iodine treatment for hyperthyroidism and thyroid cancer. *Eur J Nucl Med*.

[8] Coggle JE, Noakes GR (1983). *Biological effects of radiation - Radiation and cancer*.

[9] Holst JP, Burman KD, Atkins F, Umans JG, Jonklaas J (2005). Radioiodine therapy for thyroid cancer and hyperthyroidism in patients with end-stage renal disease on hemodialysis. *Thyroid*.

[10] Moorhead PS, Novell PC, Mellman WJ, Battips DM, Hungerford DA (1960). Chromosome preparation of leukocyte culture from peripheral blood. *Exp Cel Res*.

[11] Hungerford DA (1960). Pachytene chromosome maps of human chromosomes 19 and 20. *Cytogenet Cell Genet*.

[12] (1985). WHO. *Guidelines for the study of genetic effects in human populations*, Environmental Health Criteria, No. 46.

[13] Fenech M, Morley AA (1983). Measurement of micronuclei in human lymphocytes. *Mutat Res*.

[14] Countryman PI, Heddle JA (1976). The production of micronuclei from chromosome aberrations in irradiated cultures of human lymphocytes. *Mutat Res*.

[15] Eric Hall, Amato J (2006). Giaccia. *Radiobiology for the radiologist*.

[16] Gutiérrez S, Carbonell E, Galofré P, Creus A, Marcos R (1997). Micronuclei induction by 131I exposure.Study in hyperthyroidism patients. *Mutat Res*.

[17] Ramirez MJ, Puerto S, Galofre P, Parry EM, Parry JM, Creus A (2000). Multicolour FISH detection if radioactive iodine induced 17ce-p53 chromosomal breakages in buccal cells from therapeutically exposed patients. *Carcinogenesis*.

[18] Gutierrez S, Carbonell E, Galofre P, Creus A, Marcos R (1999). Cytogenetic damage after 131-iodine treatment for hyperthyroidism and thyroid cancer.A study using the micronucleus test. *Euro J Nuc Med*.

[19] Heddle JA, Cimino MC, Hayashi M, Romagna F, Shelby MD, Tucker JD (1991). Micronuclei as anindex of cytogenetic damage: past, present and future. *Environ Mol Mutagen*.

[20] Müller WU, Nüsse M, Miller BM, Slavotinek A, Viaggi S, Streffer C (1996). Micronuclei: a biological indicator of radiation damage. *Mutat Res*.

[21] Watanabe N, Yokoyama K, Kinuya S, Shuke N, Shimizu M, Futatsuya R (1998). Evaluation of radiotoxicity after I 131 therapy for thyroid cancer using the micronucleus assay. *J Nucl Med*.

[22] Buckton KE, Evans HJ (1973). *Methods for the analysis of human chromosome aberrations*.

